# Eating before Sleeping

**Published:** 1883-05

**Authors:** 


Eating Before Sleeping.—Man is the only animal that can be taught to sleep quietly on an empty stomach. The brute creation resent all efforts to coax them to such a violation of the laws of nature. The lion roars in the forest until he has found his prey, and when he has devoured it he sleeps over until he needs another meal. The horse will paw all night in the stable, and the pig will squeal in the pen, refusing all rest or sleep until they are fed. The animals which chew the cud have their own provision for a late meal just before dropping off to their nightly slumbers.

Man can train himself to the habit of sleeping without a preceding meal, but only after long years of practice. As he comes into the world, nature is too strong for him, and he must be fed before he will sleep. A child's stomach is small, and when perfectly filled, if no sickness disturbs it, sleep follows naturally and inevitably. As digestion goes on the stomach begins to empty. A single fold in it makes the little sleeper restless ; two will wake it ; and if it is hushed again to repose, the nap is short. Paregoric or other narcotic may close its eyes again, but without either food or some stupefying drug it will not sleep, no matter how healthy it may be. Not even an angel who learned the art of minstrelsy in a celestial choir can sing a baby to sleep upon an empty stomach. We use the oft-quoted illustration, "sleeping as sweetly as an infant," because this slumber of a child follows immediately after its stomach is completely filled with wholesome food. The sleep which comes to adults long after partaking of food, and when the stomach is nearly or quite empty, is not after the type of infantile repose. There is all the difference in the world between the sleep of refreshment and the sleep of exhaustion.

To sleep well, the blood that swells the veins in the head during our busy hours must flow back, leaving a greatly diminished volume behind the brow that lately throbbed with such vehemence. To digest well, this blood is needed at the stomach and nearer the fountain of life. It is a fact, established beyond the possibility of contradiction, that sleep aids this digestion, and that the process of digestion is conducive to refreshing sleep. It needs no argument to convince us of this mutual relation. The drowsiness which always follows the well-ordered meal is itself a testimony of nature to this inter-dependence.

